# miR-145 inhibits mitochondrial function of ovarian cancer by targeting ARL5B

**DOI:** 10.1186/s13048-020-00762-0

**Published:** 2021-01-08

**Authors:** Shuo Zhao, Yun Zhang, Meili Pei, Lei Wu, Jie Li

**Affiliations:** 1 Department of SICU, the First Affiliated Hospital of Xi’an Jiaotong University, Xi’an, China; 2 Department of Pathology, the First Affiliated Hospital of Xi’an Jiaotong University, 277 West Yanta Road, 710061 Xi’an, Shaanxi China; 3 Department of Gynecology and Obstetrics, the First Affiliated Hospital of Xi’an Jiaotong University, Xi’an, China

**Keywords:** miR-145, ARL5B, Mitochondrial function, Ovarian cancer

## Abstract

Metabolic reprogramming refers to the transformation of the whole metabolic network including glycolysis and mitochondrial metabolism, mainly manifested in Warburg effect and mitochondrial metabolic reprogramming. The roles of miR-145 in glycolysis have been established in ovarian cancer cells. Howerer, its roles in mitochondrial metabolic reprogramming are still unclear. This study aims to identify whether miR-145 regulates mitochondrial metabolic reprogramming in ovarian cancer cells. First, functional experiment showed that overexpression of miR-145 inhibited mitochondrial function in ovarian cancer cells, evident by the decreased mtDNA copy numbers, ATP level, mitochondrial membrane potential, and the expression levels of mitochondrial markers. Mechanistically, miR-145 inhibited mitochondrial function by targeting ARL5B directly. Futhermore, miR-145 overexpression decreased ARL5B expression in ovarian cancer tissue subcutaneous tumors of nude mice. In conclusion, we have highlighted that miR-145 inhibits mitochondrial function and achieves this by targeting ARL5B directly for the first time. The results provides a more adequate theoretical basis for understanding the molecular pathology of ovarian cancer, and provides the necessary basic data for miR-145 as a potential diagnosis and treatment target for ovarian cancer.

## Introduction

Ovarian cancer is one of the most common malignant tumors in women, and its mortality rate ranks first in gynecological tumors [1]. The main treatment of ovarian cancer is surgical treatment, supplemented by intravenous chemotherapy or intraperitoneal chemotherapy or combined treatment [2]. However, positive treatment did not achieve satisfactory results, the molecular mechanism and effective diagnostic biomarkers for this cancer have yet to be identified.

For many years, cancer has been regarded as an uncontrolled proliferative disease. Although metabolic changes have been studied in tumor cells, it has been considered as a secondary phenomenon. Recently, more and more evidence suggest that tumor is a primary metabolic disease [3, 4]. The metabolic research of tumor cells was first proposed by Otto Warburg, who found that most tumor cells still choose glycolysis pathway to provide energy and raw materials for biosynthesis even under aerobic conditions [5]. In fact, the complexity of the regulation of metabolic balance in tumor cells is far beyond Warburg’s original assumption [6, 7]. Increasing evidence has shown that some cancer subtypes may rely more on their intact mitochondrial respiration, which plays a pivotal role in tumor progression, and the molecular mechanisms underlying metabolic reprogramming of cancer cells has become an important topic in cancer research [8].

MicroRNAs (miRNAs) are a class of endogenous non coding small RNAs with a length of about 22 nt, which widely exist in eukaryotes [9]. miRNAs are closely related to tumors and act as oncogenes or tumor suppressor genes. It plays an important role in the growth, proliferation, differentiation, apoptosis, invasion and metastasis of tumor cells [10, 11]. We and other have shown that miR-145 is low expressed in the ovarian cancer and plays an anti-cancer role. miR-145 inhibits ovarian cancer cells migration and invasion by targeting FSCN1 and FLNB [12, 13]. inhibits glutamine metabolism through c-myc/GLS1 pathways [14], and is involved in regulation of the Warburg effect through the miR-133b/PKM2 signaling pathway [15]. However, it remains unclear whether miR-145 participates in mitochondrial metabolic reprogramming in ovarian cancer.

In the current study, functional experiments revealed that miR-145 inhibited mitochondrial function. Mechanistic research showed that miR-145 regulated the mitochondrial function through targeting ARL5B (ADP-ribosylation factorlike 5B) directly. This study provides the necessary basic data for the transformation of miR-145 to clinical application.

## Materials and methods

### Human tissue specimens

Human ovarian carcinomas and normal ovarian tissue samples were collected from patients at The First Affiliated Hospital of Xi’an Jiaotong University, China. This study was approved by the Ethics Committee of The First Affiliated Hospital of Xi’an Jiaotong University, China. Written consent was obtained from each study participant enrolled.

### Cell culture

The human ovarian cancer cell line SKOV3 was obtained from the Shanghai Cell Bank of Chinese Academy of Sciences (Shanghai, China); 3AO was obtained from the Shandong Academy of Medical Sciences (Jinan, China). Cells were maintained in RPMI 1640 medium(Gibco-BRL, Gaithersburg, MD, USA) supplemented with 10% (v/v) FBS at 37 °C under a humidified 5% CO2 atmosphere.

### microRNA mimics transfection

miR-145 mimic and control mimic were purchased from Ribo-Bio Biotechnology Co. Ltd(Guangzhou, China), and transfected instantaneously using Roche’s X-treme GENE siRNA transfection reagent(Roche, Indianapolis, IN, USA). The cells were inoculated into 6-well plates with 3 × 10^5^ holes. After 24 hours, the degree of cell fusion reached 30%-40%. SKOV3 and 3AO cells were transiently transfected with 60 nM miR-145 mimic or negative control using the X-treme GENE siRNA Transfection Reagent following the manufacturer’s protocol.

### Plasmids transfection

Human ARL5B expression vector pDEST47-ARL5B was obtained from Addgene (Boston, MA, USA). The cells were inoculated into 6-well plates with 3 × 10^5^ holes. After 48 hours, cells were until 70%-90% confluency and transiently transfected with pDEST47-ARL5B or empty vector using the X-treme GENE HP DNA Transfection Reagent (Roche, Indianapolis, IN, USA) following the manufacturer’s protocol.

### Real‐time PCR

Total RNA was extracted from cells using TRIzol reagent (Invitrogen, Carlsbad, CA, USA) according to the manufacturer’s instructions. RNA reverse transcription was performed using RevertAid first strand cDNA synthesis Kit (Thermo Fisher Scientific Inc., Waltham, MA, USA). The primer dry powder was diluted to 10 µM working concentration, and the real-time quantitative PCR using a SYBR Premix Ex Taq™ II kit (Takara, Dalian, China) on a CFX96 real-time PCR system (Bio-Rad, Hercules, CA, USA), and the reaction solution was prepared using the reverse transcription DNA as template according to the following system: SYBY Green 10µL, upstream primer 0.4µL, downstream primer 0.4µL, DNA template 2.0µL, ddH2O 7.2µL. In each experiment, a negative control group without template was set up. Each sample was set up with three multiple holes and repeated for three times. RQ values were calculated by 2^−ΔΔCt^ method to compare the differences of gene expression between each group. miR-145 were normalized to small nuclear U6, while ARL5B was normalized to the gene β-actin. The following primer sequences were used:

ARL5B forward: 5’- AGTGGGACTGGATAATGCAGGG-3’;

ARL5B reverse: 5’- ATCGCAGAGACTCCTGACCACC-3’;

β-actin forward: 5’-TCCCTGGAGAAGAGCTACGA-3’;

β-actin reverse: 5’-AGCACTGTGTTGGCGTACAG-3’.

### Western blotting

The concentration of total protein in cells was determined by the BCA-200 Protein Assay kit (Pierce, Rockford, IL, USA). Electrophoretic gels were prepared for SDS-PAGE. After electrophoresis, cut the adhesive strip to the appropriate size, balance with the membrane transfer buffer, cut the filter paper and NC membrane of the same size as the adhesive strip in advance, and immerse it in the transfer membrane buffer for 10 min. At the end of membrane transfer, the power supply was cut off and the membrane was taken out for immunoblotting. Decolorization on the shaking table of the 5% skim milk powder TBST buffer at room temperature to seal the 1H on the sulfate. Add anti-ARL5B antibody (1:500, Abcam; Cambridge, MA, USA), anti-SDHA, anti-HSP60, anti-Cytc (1:500, Cell Signaling Technology; Beverly, MA, USA), anti-β-actin (1:1000, Cell Signaling Technology; Beverly, MA, USA), incubate overnight at 4 C, add 1:2000 horseradish peroxidase labeled goat anti-rabbit antibody(Pierce Company, USA) or 1:2000 horseradish peroxidase labeled goat anti-mouse antibody (Pierce Company, USA) Incubation at room temperature for 1 hour. Electro-chemiluminescence (ECL) imaging. Blots were visualized using ECL reagents (Pierce, Rockford, IL, USA) by a chemiluminescence imaging system (Bio-Rad, Richmond, CA, USA).

### Mitochondria copy number test

1–3 µg RNA was reverse transcribed to cDNA using the RevertAid First Strand cDNA Synthesis Kit(Thermo Fisher Scientific Inc., Waltham, MA, USA). Mitochondrial DNA (mtDNA) was assessed by real-time quantitative PCR, using mitochondrial D-loop as target gene, normalized as 18S rRNA. The primer sequences for D-loop(F: 5’-AAGTGGCTGTGCAGACATTC-3’, R: 5’-TCTGTCTTTGATTCCTGCCT-3’) and 18S.

rRNA (F: 5’-TCTCCTACTTGGATAACTGTGG-3’, R: 5’-GGCGACTACCATCGAAAGTTG-3’).

### ATP assay

ATP concentrations were tested with enhanced ATP assay kit (Beyotime, Shanghai, China) according to the manusfactuer’s protocol. For adherent cells, the culture medium was removed, and 200 µL ATP was added into each well of 6-well plate to detect the proportion of lysate, and then directly added into the hole to lyse the cells. After pyrolysis, the supernatant was centrifuged at 4 ℃ for 5 min at 12,000 g. The ATP detection lysate and ATP standard solution (0.5 mM) were dissolved on ice, and the ATP standard solution was diluted into appropriate concentration gradient with ATP detection lysate. Seven concentrations were set as follows: 0.01 µM, 0.03 µM, 0.1 µM, 0.3 µM, 1 µM, 3 µM and 10 µM, respectively. According to the proportion of 100 µ l ATP detection working solution required for each sample or standard, an appropriate amount of ATP detection working fluid was prepared. Take appropriate amount of ATP detection reagent and dilute ATP detection reagent with ATP detection diluent in the ratio of 1:5. Add the prepared 100 µL ATP detection working solution into the detection tube, place it at room temperature for 3 ~ 5 min, add 20µL sample or standard substance into the detection tube, mix it quickly with a micro pipette, and measure the RLU value with a luminometer (Promega, Madison, WI, USA) after 2 s of action. ATP levels was estimated based on the standard curve, and normalized to the cell number.

### Mitochondrial membrane potential (JC-1)

The cells were digested with 0.25% trypsin without EDTA. After digestion, the cells were collected, centrifuged at 1200 rpm for 5 min, and the supernatant was suspended in 0.5 mL cell culture medium. Add 0.5 mL JC-1 dyeing working solution, invert for several times, mix well, and incubate in cell incubator at 37 ℃ for 20 minutes; during incubation, add 4 ml distilled water to every 1 mL JC-1 staining buffer (5x), and place it in ice bath. After incubation at 37 ℃, the supernatant was centrifuged at 1200 rpm 4 ℃ for 3 minutes and the supernatant was discarded, Then washed twice with JC-1 staining buffer (1x), cells were resuspended by adding 1 mL JC-1 staining buffer (1x), centrifuged at 1200 rpm for 3 minutes, and the supernatant was discarded. The cells were resuspended with 1 mL JC-1 staining buffer (1 x), centrifuged at 4 ℃ for 3 minutes at 1200 rpm, and the supernatant was discarded. After resuspension with 500 UL JC-1 staining buffer (1 x), the cells were analyzed by flow cytometry.

### Dual‐luciferase assay

miR-145 mimic, WT and pRL-TK were co-transfected into SKOV3 and 3AO cells. miR-145 mimic control, WT and pRL-TK were transfected into SKOV3 and 3AO cells as controls. After 24 hours, firefly signal and seakidney fluorescence signal were read by a dual-luciferase reporter gene assay system (Promega, Madison, WI, USA), and the relative fluorescence value of seakidney signal/firefly signal was used for comparison.

### Immunohistochemistry (IHC)

Samples were fixed in 10% neutral buffered formalin, dehydrated and embedded in paraffin. The paraffin-embedded tissues were cut into five-micron-thick serial sections. Conventional dewaxing, antigenic microwave thermal retrieval, and quenching endogenous peroxidase activity were performed on sections. Primary antibodies(anti-ARL5B antibody, 1:200; Abcam, Cambridge, MA, USA) were applied to the sections at 4 °C overnight. Next, the sections were treated with secondary antibody for 30 minutes at room temperature. Polymerase auxiliary agent was then added and incubated for another 30 minutes at room temperature and stained with 3, 3′-diaminobenzidine (DAB). Finally, the slides were counterstained with 0.02% Hematoxylin and tested by microscope.

### Statistical analysis

Each experiment was independently performed at least 3 times. The graphical presentations were performed using GraphPad Prism 5.0. Data were presented as the means ± SE and were analyzed using SPSS 22.0 software (Chicago, IL, USA). Statistical differences were tested by Chi-square test, two-tailed t-test, one-way ANOVA test or Fisher’s Exact test. A value of *P* < 0.05 was considered to be significant.

## Results

### miR-145 was inversely correlated with ARL5B in EOC

**Fig. 1:**
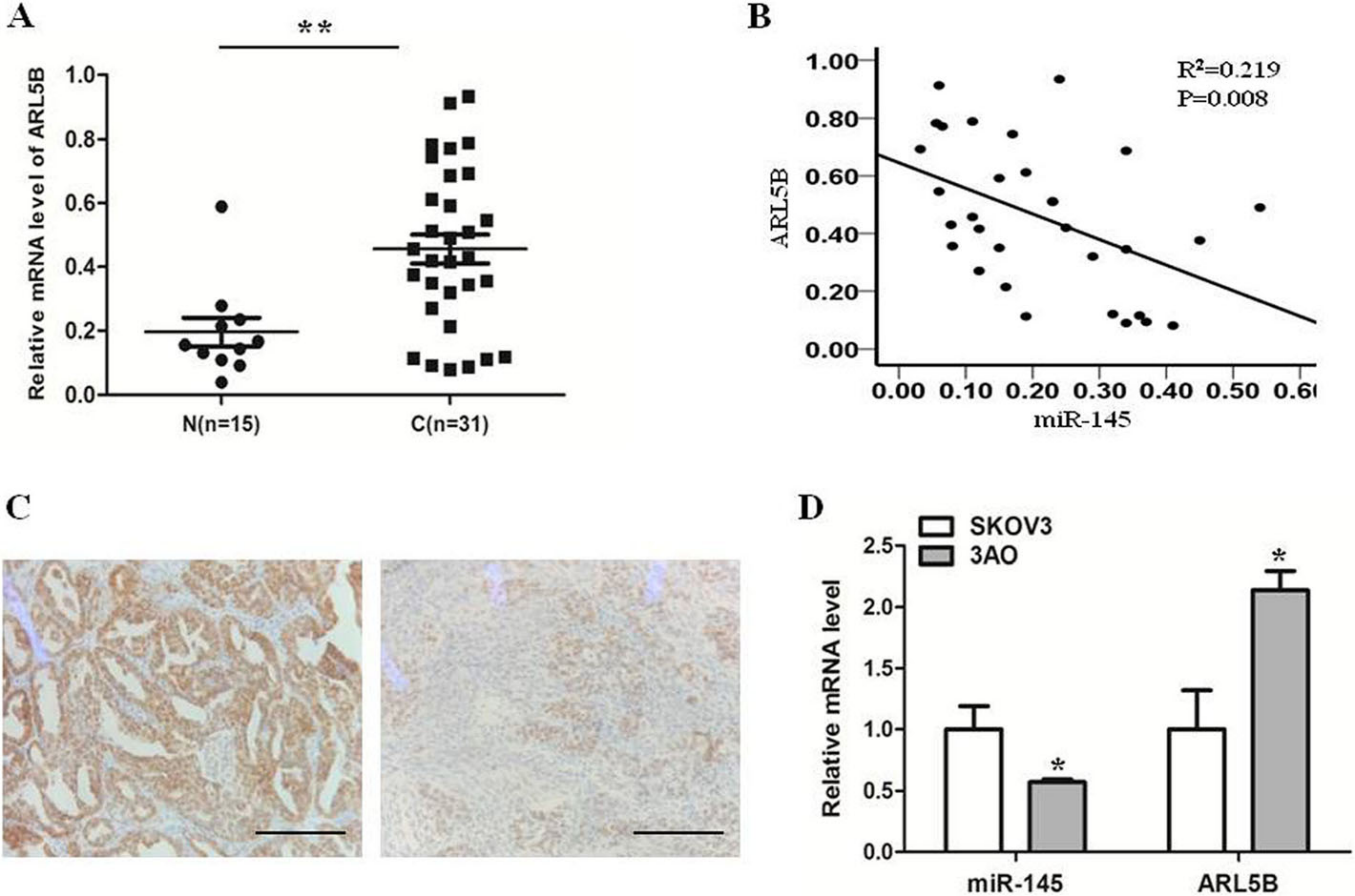
miR-145 was inversely correlated with ARL5B in EOC. **a** mRNA level of  ARL5B in ovarian cancer tissue samples (*n* = 31) was higher than in normal ovarian tissue samples (*n* = 15). **b **Scatter diagram showing ARL5B expression and miR-145 expression by qRT-PCR and their correlations (R^2^ = 0.219, *P* = 0.008) in 31 EOC tissue samples. **c **Immunohistochemistry results indicate a negative correlation between miR-145 and ARL5B in EOC epithelia. **d **Quantitative real-time PCR results show a negative correlation between ARL5B and miR-145 in both SKOV3 and 3AO cells. Scale bar, 50 µm

We detected RNA levels of miR-145 and DNMT3A in 15 normal ovarian tissue samples and 31 ovarian cancer tissue samples. In previous study [16], we have found that miR-145 level in ovarian cancer tissue samples was lower than in normal ovarian tissue samples in previous study, and we found that miR-145 level was inversely associated with clinical stage. In present study, we found ARL5B level in ovarian cancer tissue samples was higher than in normal ovarian tissue samples (Fig. 1a). Moreover, by comparing the relationship between RNA expression levels of miR-145 and ARL5B in ovarian cancer tissue samples, we found that the mRNA expression of ARL5B was negatively correlated with miR-145 in ovarian cancer tissue samples (Fig. 1b). Typical IHC photos from both miR-145^high^ and miR-145^low^ groups are shown in Fig. 1c. We identified the differential expression of miR-145 and ARL5B among EOC cell lines. The results showed that the ARL5B level in the miR-145 high-expression cell line (SKOV3) was lower than in the miR-145 low-expression cell line (3AO) (Fig. 1d).

### miR-145 inhibits mitochondrial function in ovarian cancer cells

**Fig. 2:**
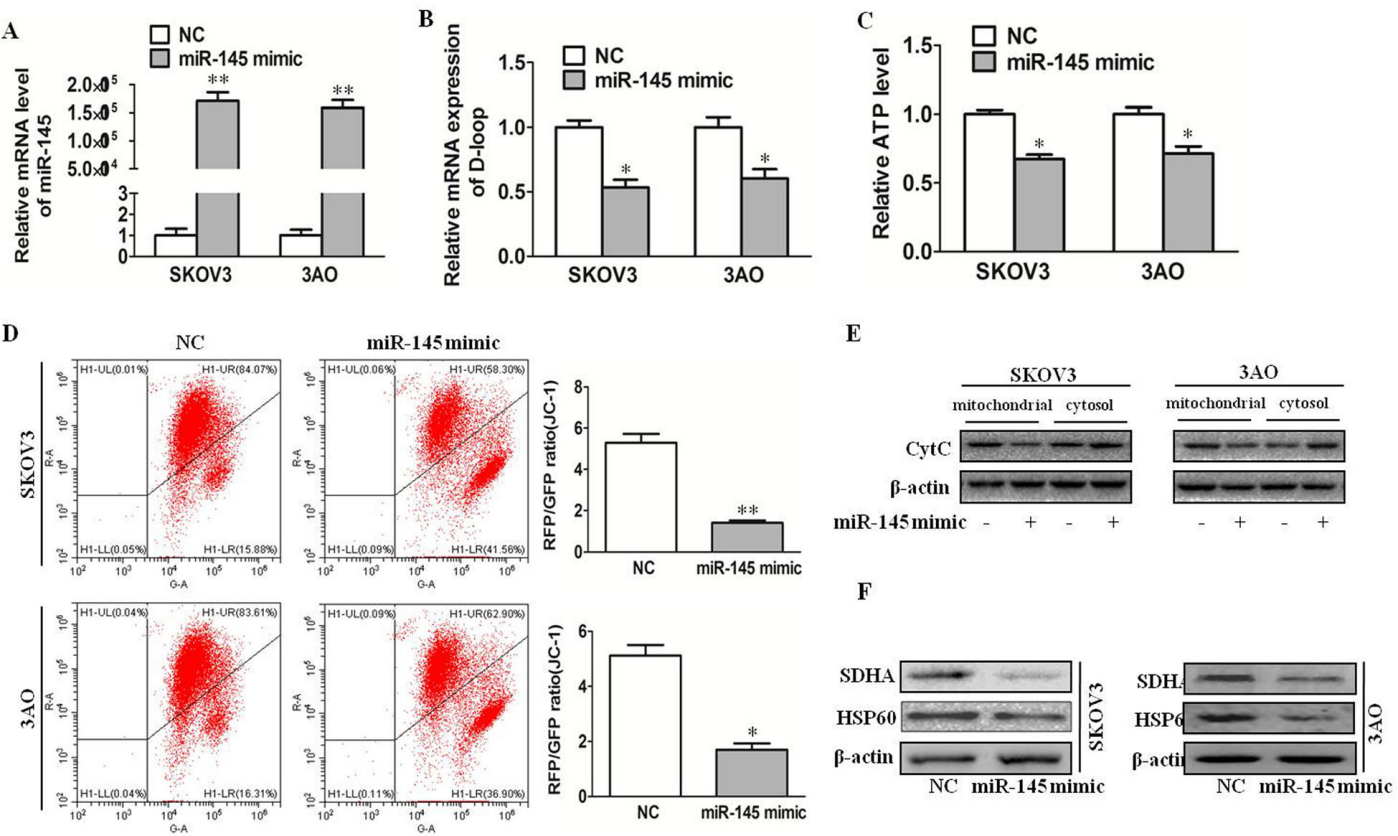
miR-145 inhibits mitochondrial function in ovarian cancer cells. **a **miR-145 expression after transfection miR-145 mimic. **b** qRT-PCR results showed overexpression of miR-145 decreased mitochondrial DNA copy numbers. **c** ATP levels in overexpression of miR-145 decreased in SKOV3 and 3AO cells. **d** Mitochondrial membrane potential analyzed by JC-1 fluorescent probe (shown as RFP/GFP ratio) in miR-145 high-expression cells reduced. **e** Release of CytC was examined in both the cytosol and mitochondrial compartments. **f** The expression levels of mitochondrial markers (SDHA and HSP60) in upregulation of miR-145 expression cells by Western blotting. *P < 0.05, **P < 0.01

To investigate whether miR-145 could regulate the mitochondrial function of ovarian cancer cells, qRT-PCR was performed to detect the expression of D-loop (control region displacement loop), which is the control gene of mitochondrial DNA replication. The results showed that overexpression of miR-145(Fig. 2a) decreased the copy number of mitochondrial DNA(Fig. 2b). Then, ATP level detection results showed that overexpression of miR-145 decreased ATP levels in ovarian cancer cells(Fig. 2c). Mitochondrial membrane potential is an important indicator of mitochondrial membrane integrity. We detected the mitochondrial membrane potential of miR-145-overexpression cells by JC-1 staining. Results as shown in Fig. 2d, overexpression of miR-145 decreased mitochondrial membrane potential. cytochrome C(CytC) is a conserved electron transport protein located in the inner space of mitochondria. Release of CytC was examined in both the cytosol and mitochondrial compartments, and we foune miR-145 increased cytochrome C release(Fig. 2e). Furthermore, the protein expression of mitochondrial markers was detected. The related markers include: SDHA, a key component of TCA cycle and electron transport chain; HSP60, which plays an important role in protein folding into mitochondria; Western blotting results demonstrated that overexpression of miR-145 down regulated the expression of SDHA and HSP60 (Fig. 2f). Taken together, these data indicated that miR-145 inhibited mitochondrial function in ovarian cancer cells.

### ARL5B is a target of miR-145

**Fig. 3:**
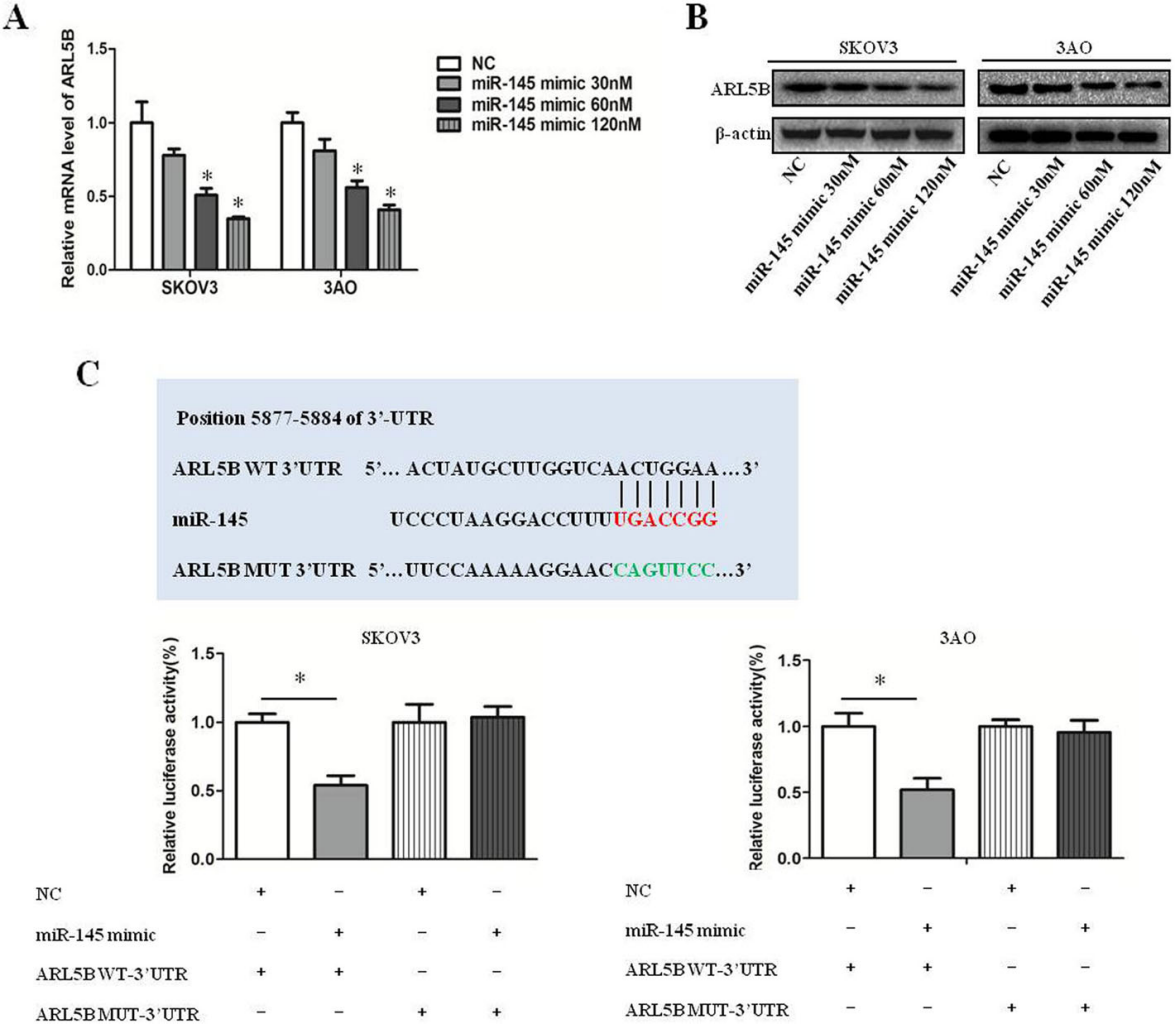
ARL5B is a target of miR-145. **a **qRT-PCR results showed overexpression of miR-145 decreased the mRNA expression of ARL5B. **b **Western blot results showed overexpression of miR-145 decreased the protein expression of ARL5B. **c **The luciferase activity of ARL5B WT and ARL5B MUT was examined in SKOV3 and 3AO cells transfected with miR-145 overexpression or not. *P < 0.05, **P < 0.01

We further detected the effect of miR-145 on the expression of ARL5B. We treated ovarian cancer cells with different concentrations of miR-145 mimic(30 nM, 60 nM, 120 nM). The results showed that the mRNA expression of ARL5B decreased in a dose-dependent manner after overexpression of miR-145(Fig. 3a), and the protein level was consistent with mRNA level(Fig. 3b). We predicted the interaction between miR-145 and 3’-UTR of ARL5B according to the TargetScan website(http://www.targetscan.org/mamm_31/) and miRBase(http://www.mirbase.org/), Then dual-luciferase reporter assay verified that miR-145 might negatively regulate ARL5B expression by directly binding to the sequence of ARL5B 3’UTR(Fig. 3c). Taken together, these data indicated that ARL5B was a target of miR-145.

### miR-145 inhibits mitochondrial function by depressing ARL5B in ovarian cancer cells

**Fig. 4:**
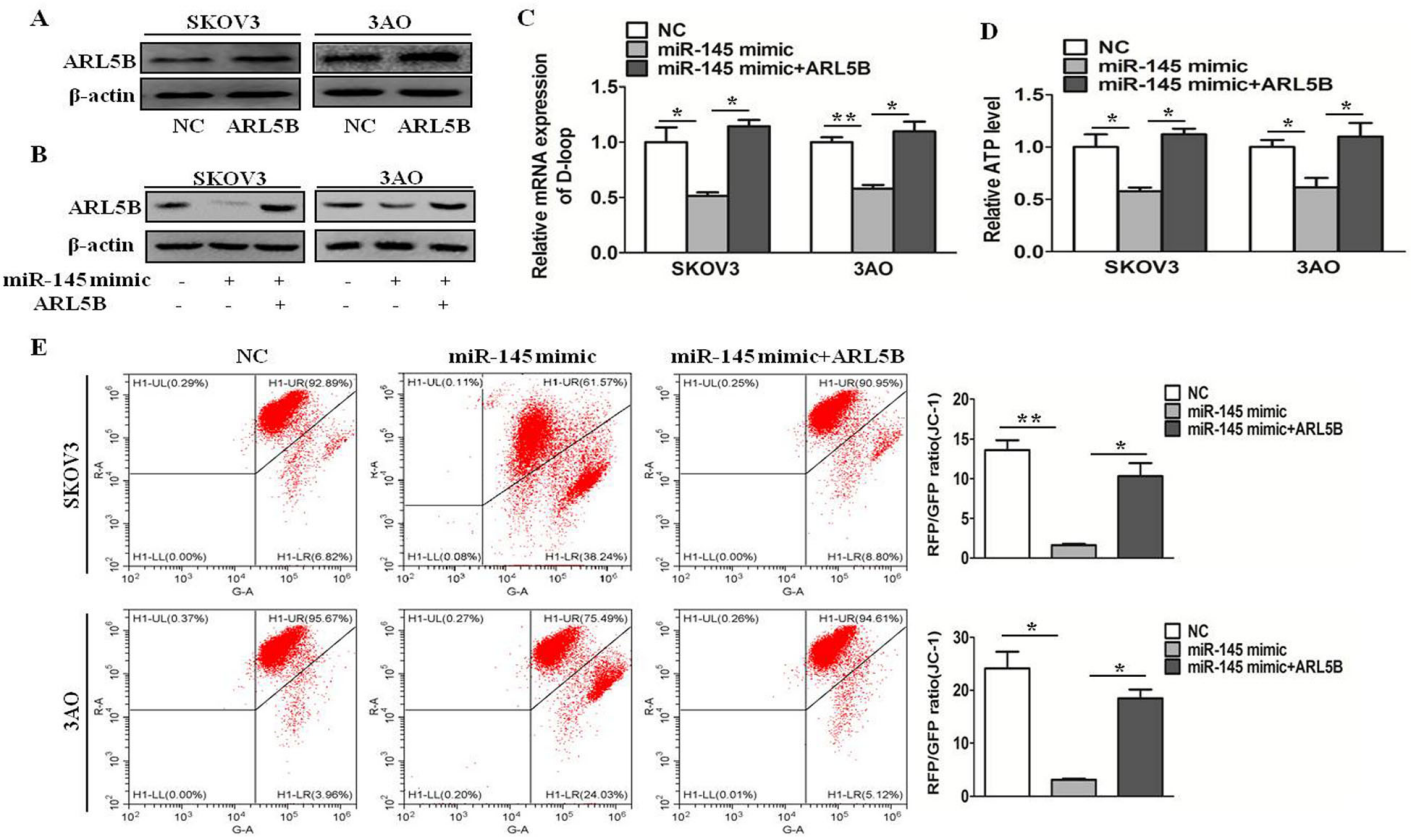
miR-145 inhibits mitochondrial function by depressing ARL5B in ovarian cancer cells. **a **ARL5B expression after transfection ARL5B plasmid. **b** ARL5B expression after transfected with NC mimic, miR-145 mimic, and miR-145 mimic + pDEST47-ARL5B. **c **Relative D-loop expression of SKOV3 and 3AO cells after transfected with NC mimic, miR-145 mimic, and miR-145 mimic + pDEST47-ARL5B. **d** Relative ATP level in different groups, including NC mimic, miR-145 mimic, and miR-145 mimic + pDEST47-ARL5B. **e** Mitochondrial membrane potential (JC-1) in different groups, including NC mimic, miR-145 mimic, and miR-145 mimic + pDEST47-ARL5B. *P < 0.05, **P < 0.01

To further clarify whether miR-145 inhibited mitochondrial function through suppressing ARL5B, we evaluated the mtDNA copy number, ATP production, and mitochondrial activity in different groups, including NC mimic, miR-145 mimic, and miR-145 mimic + ARL5B. After transfection with ARL5B plasmid, the overexpression efficiency of ARL5B was shown in Fig. 4a. The results showed overexpression of miR-145 inhibited the mtDNA copy number(Fig. 4c), ATP production(Fig. 4d), and mitochondrial activity(Fig. 4e), and after overexpression of ARL5B(Fig. 4b), this inhibitory effect was reversed. These results demonstrated that miR-145 inhibited mitochondrial function by depressing miR-145 in ovarian cancer cells.

### Expression of miR-145 and ARL5B in ovarian cancer tissue subcutaneous tumors of nude mice

**Fig. 5:**
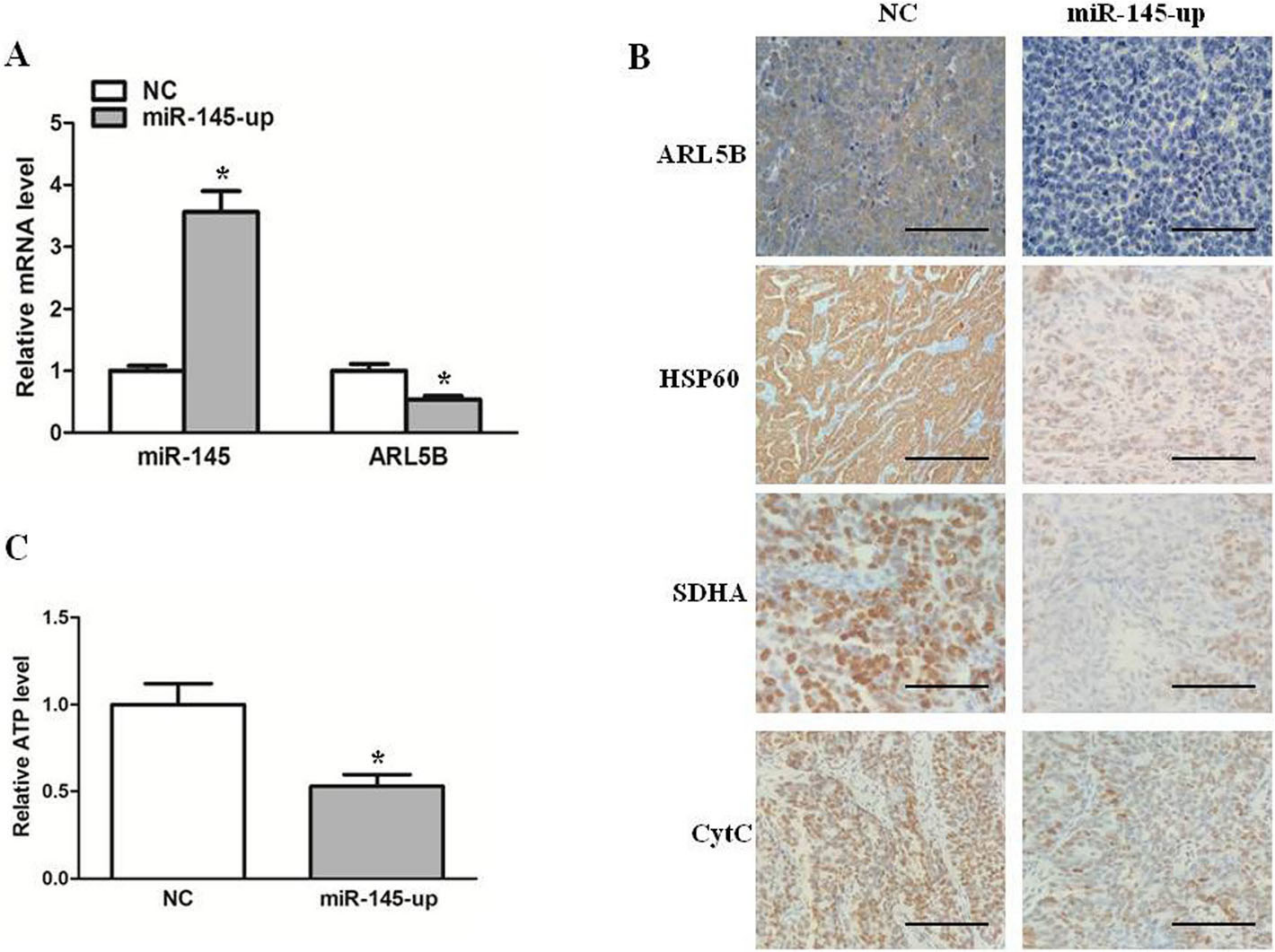
Expression of miR-145 and ARL5B in ovarian cancer tissue subcutaneous tumors of nude mice. **a** Relative mRNA expression of miR-145 and ARL5B in tumor tissues. **b **The protein expression level of ARL5B, HSP60, SDHA and CytC detected by immunochemistry staining. **c** ATP levels in xenografts of ovarian cancer. Scale bar, 50 µm *P < 0.05, **P < 0.01

In our previous studies, we found that miR-145 inhibited growth of xenografts of ovarian cancer in nude mice [12]. Here, to further investigate whether miR-145 could regulate mitochondrial function of SKOV3 cells in vivo, we analyzed the mRNA expression levels of ARL5B and miR-145 by real-time PCR, which showed decreased mRNA levels of ARL5B when miR-145 was upregulated in vivo(Fig. 5a), and immunohistochemical analysis of ARL5B, HSP60 and SDHA in miR-145-up mice was lowe than that in the control group(Fig. 5b). Futhermore, the results of immunohistochemical staining showed that the expression of CytC in cytoplasm increased after overexpression of miR-145, suggesting that miR-145 promoted the release of CytC in vivo(Fig. 5b). ATP level detection results showed that overexpression of miR-145 decreased ATP levels in xenografts of ovarian cancer(Fig. 5c). Collectively, These in vivo findings coincide with the in vitro changes observed in the cell models, showing that miR-145 inhibited ARL5B expression in ovarian cancer.

## Discussion

Metabolic reprogramming of tumor cells provides energy and material for tumor growth, invasion and metastasis, and promotes the occurrence and development of tumors. Therefore, it is considered as one of the important characteristics of tumor occurrence and development [6, 17]. Metabolic reprogramming refers to the transformation of the whole metabolic network including glycolysis and mitochondrial metabolism, mainly manifested in Warburg effect and mitochondrial metabolic reprogramming [18, 19]. In recent years, more and more studies have found that mitochondria are not bystanders in the process of tumor development, but some tumor cells are more dependent on mitochondrial metabolism [20, 21]. Mitochondrial metabolism is not only the energy metabolism center of tumor cells, but also an important hub of many biological macromolecular synthesis pathways [20, 21]. miR-145 is involved in regulation of the Warburg effect through the miR-133b/PKM2 signaling pathway [15] and targeting HK2 directly [16], which strongly suggests that miR-145 participates in metabolic reprogramming. However, the mechanism whereby miR-145 participates in tumor mitochondrial function has not been clarified.

MicroRNAs are widely distributed in eukaryotes. They can bind to the 3’-UTR of their target gene mRNA to degrade mRNA and inhibit their translation [22, 23]. Our previous findings confirmed that miR-145 inhibited ovarian cancer growth in vivo and in vitro [12]. MiR-145 blocked epithelial-mesenchymal transition of ovarian cancer cells by targeting FSCN1 [12], inhibited glutamine metabolism by targeting c-myc [14], inhibited the Warburg effect by targeting HK2 [16], and regulated RNA methylation by targeting YTHDF2 [24]. In order to test whether miR-145 was involved in the regulation of mitochondrial function, we then detected mitochondrial biogenesis by assessing the mtDNA copy number, ATP production, mitochondrial membrane potential, and mitochondrial-associated protein expression levels. The results suggested that Mir-145 may act as an oncogene and could play an important role in mitochondrial metabolism in ovarian cancer cells.

Although it has been verified that miR-145 may modulate mitochondrial metabolism in ovarian cancer, the underlying mechanism remains unclear. We predicted that ARL5B was the target gene of miR-145 by Targetscan and confirmed by dual-luciferase reporter assay. ARL5B has been recognized as a member of the ADP ribosylation factor-like (ARL) family belonging to the RAS superfamily [25–27].Previous studies have demonstrated that ARL5B overexpression facilitated lysosome motility, resulting in lysosome dispersion and accumulation at the cell periphery [28, 29]. The present research detected that decreased lysosomal outward trafficking resulted in inhibited cancer cell invasion [30]. ARL5B silencing reduced lysosome dispersion and subsequently decreased cell invasion in prostate cancer [31]. These results suggested that ARL5B plays a role in promoting cancer. However, the role of ARL5B in ovarian cancer remains unclear.

In order to investigate whether miR-145 inhibited mitochondrial function by targeting ARL5B in ovarian cancer cells, we confirmed the mtDNA copy number, ATP production, and mitochondrial activity in different groups. The results showed overexpression of miR-145 inhibited the mtDNA copy number, ATP production, and mitochondrial activity, and after overexpression of ARL5B, this inhibitory effect was reversed.

## Conclusions

In conclusion, we have highlighted that miR-145 inhibits mitochondrial function and achieves this by targeting ARL5B directly for the first time. The results provides a more adequate theoretical basis for understanding the molecular pathology of ovarian cancer, and provides the necessary basic data for miR-145 as a potential diagnosis and treatment target for ovarian cancer.

## Data Availability

The datasets during and/or analysed during the current study available from the corresponding author on reasonable request.
